# Using CHADS_2_ and CHA_2_DS_2_-VASc scores for mortality prediction in patients with chronic kidney disease

**DOI:** 10.1038/s41598-020-76098-y

**Published:** 2020-11-03

**Authors:** Po-Chao Hsu, Wen-Hsien Lee, Szu-Chia Chen, Yi-Chun Tsai, Ying-Chih Chen, Chun-Yuan Chu, Tsung-Hsien Lin, Wen-Chol Voon, Wen-Ter Lai, Sheng-Hsiung Sheu, Ho-Ming Su

**Affiliations:** 1 Division of Cardiology, Department of Internal Medicine, Kaohsiung Medical University Hospital, Kaohsiung, Taiwan; 2 Division of Nephrology, Department of Internal Medicine, Kaohsiung Medical University Hospital, Kaohsiung, Taiwan; 3 Faculty of Medicine, College of Medicine, Kaohsiung Medical University, Kaohsiung, Taiwan; 4 Department of Internal Medicine, Kaohsiung Municipal Siaogang Hospital, Kaohsiung Medical University, 482, Shan-Ming Rd., Hsiao-Kang Dist., Kaohsiung, 812 Taiwan, ROC

**Keywords:** Kidney diseases, Kidney diseases, Predictive markers, Prognostic markers

## Abstract

Chronic kidney disease (CKD) is a public health issue and is associated with high morbidity and mortality. How to identify the high-risk CKD patients is very important to improve the long-term outcome. CHADS_2_ and CHA2DS2-VASc scores are clinically useful scores to evaluate the risk of stroke in patients with atrial fibrillation. However, there was no literature discussing about the usefulness of CHADS_2_ and CHA2DS2-VASc scores for cardiovascular (CV) and all-cause mortality prediction in CKD patients. This longitudinal study enrolled 437 patients with CKD. CHADS_2_ and CHA2DS2-VASc scores were calculated for each patient. CV and all-cause mortality data were collected for long-term outcome prediction. The median follow-up to mortality was 91 (25th–75th percentile: 59–101) months. There were 66 CV mortality and 165 all-cause mortality. In addition to age and heart rate, CHADS_2_ and CHA_2_DS_2_-VASc scores (both *P* value < 0.001) were significant predictors of CV and all-cause mortality in the multivariate analysis. Besides, in direct comparison of multivariate model, basic model + CHA_2_DS_2_-VASc score had a better additive predictive value for all-cause mortality than basic model + CHADS_2_ score (*P* = 0.031). In conclusion, our study showed both of CHADS_2_ and CHA_2_DS_2_-VASc scores were significant predictors for long-term CV and all-cause mortality in CKD patients and CHA_2_DS_2_-VASc score had a better predictive value than CHADS_2_ score for all-cause mortality in direct comparison of multivariate model. Therefore, using CHADS_2_ and CHA_2_DS_2_-VASc scores to screen CKD patients may be helpful in identifying the high-risk group with increased mortality.

## Introduction

Chronic kidney disease (CKD), including end-stage renal disease (ESRD), is a public health issue in the world and is associated with high morbidity and mortality^1–4^. Cardiovascular (CV) disease is one of the leading causes of mortality in this population. Therefore, there are many programs of quality care and medical therapies developed to control the growing incidence, prevalence, and mortality for the patients with CKD^5,6^.

CHADS_2_ score is a useful scoring system to evaluate the risk of stroke in patients with atrial fibrillation (AF). In AF patients, there is a strong association between the CHADS_2_ score and the annual risk of stroke^7,8^. In addition, CHADS_2_ score was used to predict CV outcomes in the patients without AF^9–11^. Nevertheless, in recent years, CHA_2_DS_2_-VASc score has become a more useful score than CHADS_2_ score for prediction of stroke and systemic embolization in AF patients^12–14^. This new scoring system was also used to predict future CV outcome including mortality in non-AF patients^15–17^. However, there was no literature discussing about the usefulness of CHADS_2_ and CHA2DS2-VASc scores for CV and all-cause mortality prediction in the patients with CKD. Therefore, our study was aimed to evaluate the issue.

## Methods

### Study population

**Figure 1:**
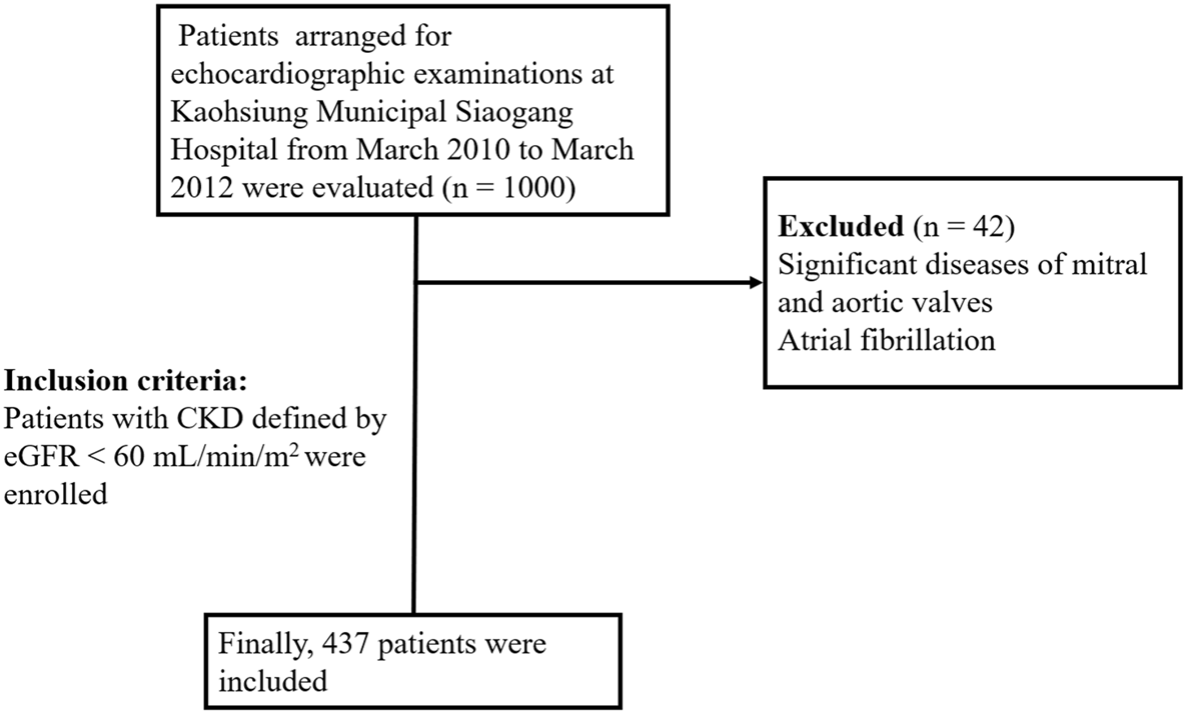
Flow chart of patient enrollment. CKD, chronic kidney disease; eGFR, estimated Glomerular filtration rate.

We evaluated a group of patients (n = 1000) arranged for echocardiographic examinations at Kaohsiung Municipal Siaogang Hospital from March 2010 to March 2012 because of suspecting coronary artery disease, hypertension, heart failure, abnormal cardiac physical examination, and survey for dyspnea. We excluded 42 subjects with significant atrial fibrillation and diseases of mitral and aortic valves. Patients with CKD defined by estimated glomerular filtration rate (eGFR) < 60 mL/min/m^2^ were enrolled. Finally, 437 patients were included (Fig. 1). This study was approved by the institutional review board committee of the Kaohsiung Medical University Hospital (KMUH-IRB). We acquired informed consents from the patients and conducted our study according to the declaration of Helsinki. We obtained medical and demographic data from the medical records.

### Assessment of CHADS_2_ score and CHA_2_DS_*2*_-VASc score

We calculated CHADS_2_ score based on the scoring system as following: 1 point was assigned for age ≧ 75 years, the presence of hypertension, diabetes mellitus, and congestive heart failure, and 2 points were assigned for transient ischemic attack or a history of stroke^7,8^. In addition, we calculated CHA_2_DS_2_-VASc score based on the scoring system as following: 1 point was assigned for congestive heart failure, hypertension, age between 65 and 74 years, diabetes mellitus, female sex, and vascular disease, and 2 points were assigned for a history of stroke and age ≥ 75 years. CKD was defined by eGFR < 60 mL/min/m^2^ and classified as stages 3, 4, and 5 based on eGFR level (30 to 59, 15 to 29, and < 15 mL/min/1.73 m^2^) with kidney damage lasting for more than 3 months.

### Definition of mortality

We followed our patients till December 2018 and acquired survival information and causes of death from the official death certificate and final confirmation by the Ministry of Health and Welfare. The causes of death were classified by the International Classification of Diseases 9th Revision. Causes of CV mortality were defined deaths due to cerebral vascular disease, ischemic heart disease, myocardial infarction, heart failure, valvular heart disease and atherosclerotic vascular disease. The detailed method was the same as our previous published paper^18^.

### Statistical analysis

SPSS 22.0 was used to perform the statistical analyses. Our data was shown as percentage or mean ± standard deviation. Categorical variables were compared by Chi-square test. Continuous variables were compared by independent samples t-test. We selected significant variables in our univariate analysis into multivariate analysis. We adjusted significant variables and time to mortality by Cox regression analysis. In addition, we also performed multivariate analysis using full model with all variables to predict the CV and all-cause mortality. We calculated the improvement of global chi-square to evaluate the additive value of CHADS_2_ score and CHA_2_DS_2_-VASc score over basic model for long-term CV and all-cause mortality prediction. Subgroup analysis by age, gender, CKD stages, hypertension, diabetes, stroke/transient ischemic attack (TIA), heart failure, and vascular disease were also performed to estimate CHADS2 and CHA2DS2-VASc score for all-cause mortality. All tests were 2-sided and the level of significance was established as *P* < 0.05.

## Results

CV and all-cause mortality data were collected up to December 2018. Mortality data were obtained from the Collaboration Center of Health Information Application (CCHIA), Ministry of Health and Welfare, Executive Yuan, Taiwan. The follow-up period to mortality events was 91 (25th–75th percentile: 59–101) months in all patients. Mortality events were documented during the follow-up period, including CV mortality (n = 66) and all-cause mortality (n = 165).

### Clinical characteristics between patients with CKD stage 3, 4, and 5

Among the 437 subjects, mean age was 68 ± 12 years. Clinical characteristics between patients with CKD stage 3, 4, and 5 were shown in Table 1. There were significant difference between different CKD stage in prevalence of diabetes (*P* = 0.001), hypertension (*P* = 0.002), stroke/TIA (*P* = 0.014), CHADS_2_ score (1.63 ± 1.15 versus 2.00 ± 1.07 versus 2.34 ± 1.30, *P* = 0.001), CHA_2_DS_2_-VASc score (2.97 ± 1.59 versus 3.46 ± 1.76 versus 3.54 ± 1.87, *P* = 0.029), calcium channel blocker use (*P* = 0.003), and diuretic use (*P* = 0.002).

**Table 1:** Comparison of clinical characteristics between patients with and without mortality.

Baseline characteristics	CKD stage 3	CKD stage 4	CKD stage 5	*P* value
Number	352	50	35	
Age (years)	68 ± 12	69 ± 13	63 ± 15	0.066
Male gender (%)	51.4%	52.0%	51.5%	0.997
Smoking (%)	10.8%	6.0%	8.6%	0.547
Diabetes (%)	32.1%	52.0%	57.1%	0.001
Hypertension (%)	72.4%	88.0%	94.3%	0.002
Dyslipidemia (%)	42.2%	55.0%	31.0%	0.128
Stroke/TIA (%)	7.1%	4.0%	20.0%	0.014
Heart failure (%)	11.9%	14.0%	14.3%	0.859
Heart rate (min^−1^)	69 ± 13	69 ± 11	74 ± 14	0.159
Body mass index	26.1 ± 4.0	26.0 ± 3.9	26.4 ± 5.9	0.897
CHADS_2_ score	1.63 ± 1.15	2.00 ± 1.07	2.34 ± 1.30	0.001
CHA_2_DS_2_-VASc score	2.97 ± 1.59	3.46 ± 1.76	3.54 ± 1.87	0.029
**Medication**				
Aspirin	35.9%	34.7%	22.9%	0.303
β-blockers	44.7%	46.0%	45.7%	0.981
CCBs	44.6%	62.0%	68.6%	0.003
ACEIs	8.2%	8.0%	5.7%	0.871
ARBs	54.0%	62.0%	48.6%	0.434
Diuretics	34.2%	50.0%	60.0%	0.002

*ACEI* angiotensin converting enzyme inhibitor, *ARB* angiotensin II receptor blocker, *CCB* calcium channel blocker, *CKD* chronic kidney disease, *TIA* transient ischemic attack.

### Predictors of CV and all-cause mortality in the univariate analysis

Several parameters were evaluated in our study to predict the CV and all-cause mortality. These parameters included age, gender, dyslipidemia, smoking, heart rate, body mass index, CHADS_2_ score, CHA_2_DS_2_-VASc scores, and medication use such as aspirin, beta blocker, calcium channel blocker, angiotensin converting enzyme inhibitor, angiotensin II receptor blocker, and diuretic. The predictors of CV and all-cause mortality using Cox proportional hazards model in the univariate analysis were shown in Table 2. For prediction of CV mortality, age, heart rate, body mass index, CHADS_2_ score, and CHA_2_DS_2_-VASc score (both *P* value < 0.001) were significant predictors. For prediction of all-cause mortality, age, heart rate, body mass index, CHADS_2_ score, and CHA_2_DS_2_-VASc score (both *P* value < 0.001), and diuretic use were significant predictors.

**Table 2:** Predictors of CV and all-cause mortality using Cox proportional hazards model (univariate analysis).

Parameter	Univariate (CV mortality)		Univariate (all-cause mortality)	
	HR (95% CI)	*P*	HR (95% CI)	*P*
Age (per 1 year)	1.067 (1.040–1.094)	< 0.001	1.073 (1.055–1.091)	< 0.001
Male gender (male vs female)	1.119 (0.682–1.836)	0.657	1.053 (0.771–1.439)	0.745
Diabetes (%)	2.330 (1.419–3.826)	0.001	1.861 (1.361–2.546)	< 0.001
Hypertension (%)	0.716 (0.421–1.217)	0.217	1.015 (0.707–1.458)	0.934
Dyslipidemia (yes or no)	0.958 (0.542–1.695)	0.883	0.760 (0.532–1.084)	0.130
Stroke/TIA (%)	3.300 (1.675–6.502)	0.001	2.795 (1.779–4.391)	< 0.001
Heart failure (%)	4.736 (2.732–8.210)	< 0.001	3.474 (2.390–5.048)	< 0.001
Smoking (ever vs no)	0.932 (0.402–2.161)	0.932	0.860 (0.497–1.488)	0.589
Heart rate (per beat/minute)	1.022 (1.003–1.040)	0.020	1.016 (1.004–1.028)	0.009
Body mass index	0.920 (0.859–0.986)	0.018	0.930 (0.891–0.971)	0.001
CHADS_2_ score	1.785 (1.478–2.157)	< 0.001	1.716 (1.521–1.936)	< 0.001
CHA_2_DS_2_-VASc score	1.661 (1.434–1.925)	< 0.001	1.611 (1.467–1.768)	< 0.001
**Medications**				
Aspirin use	1.132 (0.674–1.902)	0.639	1.154 (0.833–1.600)	0.388
Beta blocker use	1.209 (0.738–1.982)	0.451	0.992 (0.725–1.358)	0.960
Calcium channel blocker use	0.992 (0.605–1.627)	0.975	0.936 (0.684–1.279)	0.677
ACEI use	0.785 (0.285–2.162)	0.640	1.313 (0.783–2.202)	0.301
ARB use	1.104 (0.670–1.819)	0.698	0.849 (0.622–1.160)	0.305
Diuretic use	1.318 (0.797–2.177)	0.282	1.733 (1.268–2.368)	0.001

*HR* hazard ratio, *CI* confidence interval, *CV* cardiovascular, other abbreviations as in Table 1.

### Predictors of CV mortality in the multivariate analysis

We selected significant variables in our univariate analysis into multivariate analysis and used Cox proportional hazards model to evaluate the predictors of CV mortality. We tried to evaluate the predictive value of CHADS_2_ score and CHA_2_DS_2_-VASc score in two different models, respectively. Data was shown in Table 3. Model 1 included the significant variables in the univariate analysis except CHA_2_DS_2_-VASc score, including age, heart rate, body mass index, and CHADS_2_ score. Model 2 included the significant variables in the univariable analysis except CHADS_2_ score, including age, heart rate, body mass index, and CHA_2_DS_2_-VASc score. In model 1, age, heart rate, and CHADS2 score (hazard ratio [HR] = 1.574; 95% confidence interval [CI]: 1.264–1.961; *P* < 0.001) were significant predictors after multivariate analysis. In model 2, age, heart rate, and CHA_2_DS_2_-VASc score (HR 1.511; 95% CI 1.266–1.804; *P* < 0.001) were significant predictors after multivariate analysis.

**Table 3:** Predictors of CV mortality using Cox proportional hazards model (multivariate analysis).

Parameter	Model 1		Model 2	
	HR (95% CI)	*P*	HR (95% CI)	*P*
Age (per 1 year)	1.054 (1.026–1.082)	< 0.001	1.041 (1.012–1.071)	0.005
Heart rate (per beat/min)	1.029 (1.008–1.049)	0.005	1.031 (1.011–1.052)	0.003
Body mass index	–	–	–	–
CHADS_2_ score	1.574 (1.264–1.961)	< 0.001	–	–
CHA_2_DS_2_-VASc score	–	–	1.511 (1.266–1.804)	< 0.001

*HR* hazard ratio, *CI* confidence interval, *CV* cardiovascular, other abbreviations as in Table 1.

### Predictors of all-cause mortality in the multivariate analysis

We further used Cox proportional hazards model to evaluate the predictors of all-cause mortality and the data was shown in Table 4. Similar methodology was used as in Table 3. Model 1 included the significant variables in the univariable analysis except CHA_2_DS_2_-VASc score, including age, heart rate, body mass index, diuretic use, and CHADS_2_ score. Model 2 included the significant variables in the univariable analysis except CHADS_2_ score, including age, heart rate, body mass index, diuretic use, and CHA_2_DS_2_-VASc score. In model 1, age, heart rate, and CHADS_2_ score (HR 1.470; 95% CI 1.276–1.693; *P* < 0.001) were significant predictors after multivariable analysis. In model 2, age, heart rate, and CHA_2_DS_2_-VASc score (HR 1.421; 95% CI 1.266–1.596; *P* < 0.001) were significant predictors after multivariable analysis.

**Table 4:** Predictors of all-cause mortality using Cox proportional hazards model (multivariate analysis).

Parameter	Model 1		Model 2	
	HR (95% CI)	*P*	HR (95% CI)	*P*
Age (per 1 year)	1.062 (1.044–1.080)	< 0.001	1.051 (1.032–1.070)	< 0.001
Heart rate (per beat/min)	1.023 (1.010–1.036)	0.001	1.025 (1.012–1.038)	< 0.001
Body mass index	–	–	–	–
Diuretic use	–	–	–	–
CHADS_2_ score	1.470 (1.276–1.693)	< 0.001	–	–
CHA_2_DS_2_-VASc score	–	–	1.421 (1.266–1.596)	< 0.001

*HR* hazard ratio, *CI* confidence interval, other abbreviations as in Table 1.

### Predictors of CV and all-cause mortality using full model with all variables in multivariate analysis

In addition to use significant variables in the univariate analysis to perform multivariate analysis, we also performed a full model with all variables presented in Table 1 to evaluate the predictors of CV and all-cause mortality and the data were shown in Table 5. For prediction of CV mortality, after adjusting all variables, age, male gender, hypertension, heart failure, and CHA_2_DS_2_-VASc score (HR 1.600; 95% CI 1.254–2.040; *P* < 0.001) were significant predictors of CV mortality. CHADS_2_ score became non-significant after multivariate analysis (*P* 0.260). For prediction of all-cause mortality, after adjusting all variables, age, male gender, heart failure, CHA_2_DS_2_-VASc score (HR 1.503; 95% CI 1.300–1.739; *P* < 0.001), and ARB use were significant predictors of all-cause mortality. CHADS_2_ score became non-significant after multivariate analysis (*P* = 0.607).

**Table 5:** Predictors of CV and all-cause mortality using full model with all variables (multivariate analysis).

Parameter	Multivariate (CV mortality)		Multivariate (all-cause mortality)	
	HR (95% CI)	*P*	HR (95% CI)	*P*
Age (per 1 year)	1.045 (1.010–1.082)	0.011	1.055 (1.033–1.077)	< 0.001
Male gender (male vs female)	2.119 (1.157–3.882)	0.015	1.527 (1.061–2.197)	0.023
Diabetes (%)	–	0.443	–	0.619
Hypertension (%)	0.387 (0.181–0.826)	0.014	–	0.059
Dyslipidemia (yes or no)	–	0.119	–	0.681
Stroke/TIA (%)	–	0.855	–	0.950
Heart failure (%)	2.510 (1.156–5.450)	0.020	2.312 (1.407–3.800)	0.001
Smoking (ever vs no)	0.932 (0.402–2.161)	0.932	–	0.862
Heart rate (per beat/minute)	–	0.511	–	0.207
Body mass index	–	0.970	–	0.926
CHADS_2_ score	–	0.260	–	0.607
CHA_2_DS_2_-VASc score	1.600 (1.254–2.040)	< 0.001	1.503 (1.300–1.739)	< 0.001
**Medications**				
Aspirin use	–	0.796	–	0.895
Beta blocker use	–	0.629	–	0.834
Calcium channel blocker use	–	0.788	–	0.636
ACEI use	–	0.706	–	0.568
ARB use	–	0.524	0.506 (0.342–0.748)	0.001
Diuretic use	–	0.206	–	0.125

*HR* hazard ratio, *CI* confidence interval, *CV* cardiovascular, other abbreviations as in Table 1.

### Subgroup analysis in estimating CHADS2 and CHA2DS2-VASc score for all-cause mortality

We further used subgroup analysis to estimate CHADS_2_ and CHA_2_DS_2_-VASc score for all-cause mortality (Table 6). Several subgroup analysis were performed, including age (age < 65 year or ≥ 65 year), gender (male or female), CKD stage (stage 3 or stage 4–5), hypertension (yes or No), diabetes (yes or no), stroke/TIA (yes or no), heart failure (yes or no), and vascular disease (yes or no). CHADS_2_ score only showed non-significant finding in subgroup with stroke/TIA and subgroup with heart failure. CHA_2_DS_2_-VASc score only showed non-significant finding in subgroup with stroke/TIA. However, these non-significant findings might be related to small subgroup sample size. There were only 34 patients with stroke/TIA and 54 patients with heart failure. In addition, for subgroup of CKD stage, we combined CKD stage 4 (n = 50) and stage 5 (n = 35) because of small sample size (Table 6).

**Table 6:** Subgroup analysis in estimating CHADS_2_ and CHA_2_DS_2_-VASc score for all-cause mortality.

Subgroup	CHADS_2_ score		CHA_2_DS_2_-VASc score	
	HR (95% CI)	*P*	HR (95% CI)	*P*
**Age**				
Age < 65 y/o	2.339 (1.634–3.349)	< 0.001	1.796 (1.344–2.400)	< 0.001
Age > 65 y/o	1.364 (1.167–1.594)	< 0.001	1.409 (1.247–1.591)	< 0.001
**Gender**				
Male	1.332 (1.101–1.611)	0.003	1.438 (1.258–1.644)	< 0.001
Female	1.565 (1.262–1.939)	< 0.001	1.765 (1.533–2.031)	< 0.001
**CKD stage**				
Stage 3	1.493 (1.250–1.783)	< 0.001	1.643 (1.462–1.846)	< 0.001
Stage 4–5	1.352 (1.078–1.696)	0.009	1.311 (1.119–1.536)	0.001
**Hypertension**				
Yes	1.617 (1.364–1.917)	< 0.001	1.513 (1.320–1.735)	< 0.001
No	1.907 (1.305–2.789)	< 0.001	1.993 (1.617–2.456)	< 0.001
**Diabetes**				
Yes	1.349 (1.054–1.727)	< 0.001	1.415 (1.205–1.661)	< 0.001
No	1.821 (1.342–2.471)	< 0.001	1.727 (1.338–2.230)	< 0.001
**Stroke/TIA**				
Yes	–	0.097	–	0.052
No	1.413 (1.150–1.735)	< 0.001	1.401 (1.198–1.638)	< 0.001
**Heart failure**				
Yes	–	0.177	1.204 (1.019–1.423)	0.029
No	1.416 (1.191–1.684)	< 0.001	1.413 (1.218–1.639)	< 0.001
**Vascular disease**				
Yes	1.327 (1.077–1.634)	0.008	1.335 (1.102–1.618)	0.003
No	1.520 (1.237–1.867)	< 0.001	1.356 (1.126–1.632)	0.001

*HR* hazard ratio, *CI* confidence interval, other abbreviations as in Table 1.

### Nested Cox model for CV mortality and all-cause mortality prediction

**Figure 2:**
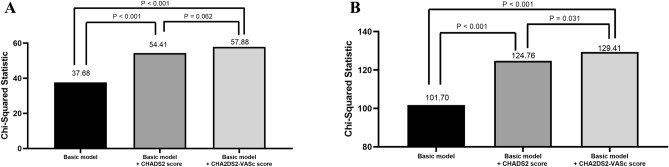
Nested Cox model for cardiovascular mortality (**A**) and all-cause mortality (**B**). Basic model in (**A**) included age, heart rate, and body mass index. Basic model in (**B**) included age, heart rate, body mass index, and diuretic use.

We used Nested Cox model for CV mortality (Fig. 2A) and all-cause mortality (Fig. 2B) prediction. We calculated the improvement of global chi-square to evaluate the additive value of CHADS_2_ score and CHA_2_DS_2_-VASc score over basic model for long-term CV and all-cause mortality prediction. The basic model in Fig. 2A included age, heart rate, and body mass index. After adding CHADS_2_ score and CHA_2_DS_2_-VASc score into the basic model respectively, we found both of basic model + CHADS_2_ score and basic model + CHA_2_DS_2_-VASC score had a better predictive value for CV mortality than basic model itself (both *P* < 0.001). However, there was no significant difference between basic model + CHADS_2_ score and basic model + CHA_2_DS_2_-VASc score (*P* = 0.062). The basic model in Fig. 2B included age, heart rate, body mass index, and diuretic use. After adding CHADS_2_ score and CHA_2_DS_2_-VASc score into the basic model respectively, we found both of basic model + CHADS_2_ score and basic model + CHA_2_DS_2_-VASc score had a better predictive value for all-cause mortality than basic model itself (both *P* < 0.001). In addition, basic model + CHA_2_DS_2_-VASc score had a better predictive value for all-cause mortality than basic model + CHADS_2_ score (*P* = 0.031).

### The Kaplan–Meier curves of different CKD stages for all-cause mortality-free survival prediction

**Figure 3:**
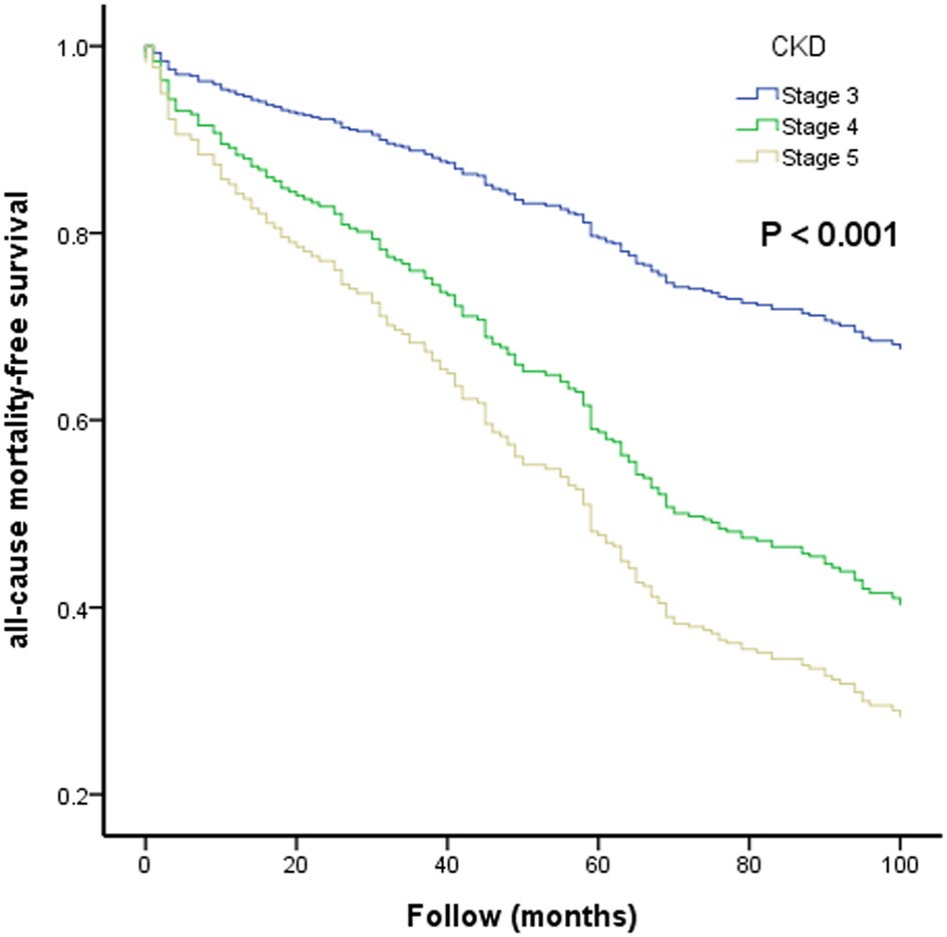
The Kaplan–Meier curves of different chronic kidney disease (CKD) stages for all-cause mortality-free survival.

We further compared the different CKD stages (CKD stage 3, 4, and 5) for all-cause mortality prediction (Fig. 3, *P* < 0.001). HR of CKD stage 4 versus stage 3 was 1.849 (95% CI 1.528–3.523; *P* < 0.001) and HR of CKD stage 5 versus stage 3 was 3.221 (95% CI 2.064–5.029; *P* < 0.001) for prediction of all-cause mortality.

## Discussion

Our study was aimed to evaluate the usefulness of CHADS_2_ and CHA_2_DS_2_-VASc scores on the prediction of CV and all-cause mortality in CKD patients. There were several major findings in the present study. First, both of CHADS_2_ and CHA_2_DS_2_-VASc scores were significant predictors of CV and all-cause mortality after multivariable analysis. Second, both of CHADS_2_ and CHA_2_DS_2_-VASc scores had an additive value than conventional parameters for prediction of CV and all-cause mortality. Furthermore, in direct comparison of multivariate model, CHA_2_DS_2_-VASc score had a better value than CHADS_2_ score for prediction of all-cause mortality, but not CV mortality. Third, higher stage of CKD was associated with higher all-cause mortality in CKD patients.

CKD was associated with accelerated risk and high event rate of CV disease, and was considered as a CV disease equivalent^19^. Patients with CKD had several risk factors that were related to atherosclerosis, such as hypertension, diabetes mellitus, dyslipidemia, smoking, and so on^20^. These risk factors could cause remodeling of the myocardium and blood vessels and lead to arterial stiffness and atherosclerosis, cardiomyopathy, and subsequently to ischemic heart disease, heart failure, CV death, rapid deterioration of renal function, and finally progression to ESRD^19^. The mortality rates associated with CKD were striking. According to the literature, mortality in patients with CKD was 56% greater than that in patients without CKD, the risk was even much higher in patients with CKD stages 4–5. For the patients with ESRD, the 5-year survival rate was only 35% in the United States^21^. Therefore, how to identify the high-risk CKD patients with increased mortality was very important to improve the long-term outcome.

Both of CHADS_2_ and CHA_2_DS_2_-VASc scores were practical and useful scoring system to evaluate the risk of stroke in AF patients^7,8,12–14^. However, CHA_2_DS_2_-VASc score had recently become a more useful score and outperformed CHADS_2_ score for prediction of stroke and systemic embolization^12,14^. In addition, both of CHADS_2_ and CHA_2_DS_2_-VASc scores were also used to predict CV outcomes in non-AF patients^9–11,15–17^. Chen et al. reported that CHADS_2_ and CHA_2_DS_2_-VASc scores could be used to predict 1-year all-cause mortality in patients with systolic heart failure^15^. Hoshino T et al. showed that CHADS_2_ and CHA_2_DS_2_-VASc scores were useful in predicting functional status after stroke in patients with coronary artery disease^16^. Svendsen JH et al. also revealed that CHADS_2_ and CHA_2_DS_2_-VASc scores were associated with increased risk of stroke and death in patients paced for sick sinus syndrome^17^. However, there was no literature discussing about the usefulness of CHADS_2_ and CHA2DS2-VASc scores for CV and all-cause mortality prediction in the patients with CKD. Our study was the first study tried to investigate the issue. In our study, both of CHADS_2_ and CHA_2_DS_2_-VASc scores were associated with increased CV and all-cause mortality in univariable and multivariable analyses. In addition, we found that CHA_2_DS_2_-VASc score had a better value than CHADS2 score for prediction of all-cause mortality in direct comparison of multivariate model (*P* = 0.031), but this finding was not found in CV mortality (*P* = 0.062). Our study also showed that increased CKD stage was associated with higher all-cause mortality, which was reasonable as our clinical practice.

### Study limitations

First, non-fatal events were not evaluated in this study. Second, CV medications might affect the study results; however, we already adjusted the medications in our multivariate analysis as possible as we can to avoid the influence of medications. Because we initially excluded the patients with atrial fibrillation, we did not collect the information of oral anticoagulant use in our study.

## Conclusions

Our study was the first study to evaluate the usefulness of CHADS_2_ and CHA_2_DS_2_-VASc scores in CKD patients for prediction of long-term CV and all-cause mortality. Our study showed both of CHADS_2_ and CHA_2_DS_2_-VASc scores were significant predictors for long-term CV and all-cause mortality in CKD patients and CHA_2_DS_2_-VASc score had a better predictive value than CHADS_2_ score for all-cause mortality in direct comparison of multivariate model. Therefore, using CHADS_2_ and CHA_2_DS_2_-VASc scores to screen CKD patients may help physicians to identify the high-risk group with increased mortality.
